# The Prescription of Drugs That Inhibit Organic Anion Transporters 1 or 3 Is Associated with the Plasma Accumulation of Uremic Toxins in Kidney Transplant Recipients

**DOI:** 10.3390/toxins14010015

**Published:** 2021-12-25

**Authors:** Camille André, Touria Mernissi, Gabriel Choukroun, Youssef Bennis, Saïd Kamel, Sophie Liabeuf, Sandra Bodeau

**Affiliations:** MP3CV Laboratory, EA7517, Jules Verne University of Picardie, F-80000 Amiens, France; andre.camille@chu-amiens.fr (C.A.); mernissi.touria@chu-amiens.fr (T.M.); choukroun.gabriel@chu-amiens.fr (G.C.); bennis.youssef@chu-amiens.fr (Y.B.); kamel.said@chu-amiens.fr (S.K.); liabeuf.sophie@chu-amiens.fr (S.L.)

**Keywords:** uremic toxin, organic anion transporter, OAT1 inhibitor, OAT3 inhibitor, kidney transplantation

## Abstract

The renal elimination of uremic toxins (UTs) can be potentially altered by drugs that inhibit organic anion transporters 1/3 (OAT1/OAT3). The objective of the present study was to determine whether the prescription of at least one OAT1/OAT3 inhibitor was associated with the plasma accumulation of certain UTs in kidney transplant recipients. We included 403 kidney transplant recipients. For each patient, we recorded all prescription drugs known to inhibit OAT1/OAT3. Plasma levels of four UTs (trimethylamine N-oxide (TMAO), indole acetic acid (IAA), para-cresylsulfate (pCS), and indoxylsulfate (IxS) were assayed using liquid chromatography-tandem mass spectrometry. Plasma UT levels were significantly higher among patients prescribed at least one OAT inhibitor (*n* = 311) than among patients not prescribed any OAT inhibitors (*n* = 92). Multivariate analysis revealed that after adjustment for age, estimated glomerular filtration rate (eGFR), plasma level of albumin and time since transplantation, prescription of an OAT1/OAT3 inhibitor was independently associated with the plasma accumulation of pCS (adjusted odds ratio (95% confidence interval): 2.11 (1.26; 3.61]). Our results emphasize the importance of understanding the interactions between drugs and UTs and those involving UT transporters in particular.

## 1. Introduction

Uremic toxins (UTs) are solutes that accumulate in patients with chronic kidney disease (CKD) as their renal function deteriorates. These heterogeneous compounds are classified into three categories as a function of their molecular weight and their ability to bind to plasma proteins: (i) small solutes with a molecular weight below 500 Da (such as trimethyl-amine-N-oxide (TMAO)), (ii) middle molecules with a molecular weight above 500 daltons (such as beta-2-microglobulin), and (iii) protein-bound UTs (PBUTs, such as indoxyl sulfate (IxS), para-cresyl sulfate (pCS), and indole acid acetic (IAA)) [1]. Although small solutes and (to a lesser extent) middle molecules can be easily removed by conventional dialysis, PBUTs are difficult to eliminate from the plasma of patients with CKD [2]. Thus, blood PBUT concentrations rise progressively as CKD worsens. The accumulation of UTs in the CKD patients’ blood and tissues induces a “uremic syndrome”, with a combination of cardiotoxicity [3,4], neurotoxicity [5], nephrotoxicity, and/or bone disease [6].

Stage 5 (end-stage) CKD is a life-threatening medical emergency requiring dialysis or kidney transplantation. Transplantation is the preferential treatment, with a higher 5-year survival vs. patients who stay on hemodialysis [7]. The estimated glomerular filtration rate (eGFR) partly recovers after transplantation, which decreases plasma PBUT concentrations [8]. Kidney transplant recipients take a mean ± standard deviation of 11 ± 3 drugs and thus have a higher, more complex drug burden than hemodialyzed patients [9]. This polypharmacy results in a greater frequency of adverse drug reactions [10] and thus might increase pharmacokinetic interactions between drugs and UTs. These interactions can involve drug transporters. The solute carrier 22 (SLC22) family of transporters (also known as organic anion transporters (OATs)) have been extensively studied because of their role in the transport of common drugs (cidofovir, methotrexate, olmesartan, etc.), toxins (mercury, ochratoxin A, etc.) and endogenous molecules (prostaglandins, vitamins, steroids, etc.) [11]. OATs are expressed in many tissues, including the kidney, liver, and brain. Many PBUTs are substrates for OAT1 (also known as SLC22A6) or OAT3 (SLC22A8) expressed on the basolateral membrane of renal proximal tubular cells. These two transporters are involved in PBUT secretion from the blood into the urine. The PBUTs interact to a variable extent with OAT1/OAT3. For example, TMAO and IxS bind with greater affinity to OAT3 than to OAT1, while pCS interacts to the same extent with both [12]. Given that UTs are OAT1/OAT3 substrates, drugs that inhibit these transporters might modify the toxicokinetics of UTs; the resulting accumulation of UTs in the blood and tissues would worsen the uremic syndrome. There are few published data on the potential impact of drug—UT interactions on UT levels. Luo et al. observed an increment in the area under the curve (AUC) and elimination half-life for IxS after administration of the OAT3 inhibitor antibiotic ciprofloxacin in nephrectomized rats [13]. Another clinical study conducted in CKD patients showed that patients treated with the OAT3 inhibitor furosemide presented a plasma and tissular accumulation of TMAO [14].

However, to the best of our knowledge, there is no clinical study evaluating the effect of global drug prescriptions of OAT1/OAT3 inhibitors on the levels of UT. The primary objective of the present clinical study was to determine whether commonly prescribed drugs that inhibit OAT1/OAT3 inhibitors are associated with differences in plasma UT levels in kidney transplant recipients.

## 2. Results

### 2.1. Baseline Characteristics

A total of 403 patients were included in the study. The population was divided into a group of patients who had not taken any OAT1/OAT3 inhibitors (*n* = 92) and a group of patients who had taken at least one OAT1/OAT3 inhibitor (*n* = 311) (Table 1). The median (IQR) age was 56 (48; 66) years, and there were 250 men (62%). The median BMI was 26.5 (23.4–29.7) kg/m^2^, and the median for time since transplantation was 78 (40.0; 158.5) months. Almost all patients (97.3%) presented hypertension. The majority of the patients (56.5%) were at CKD stage 3, with a median eGFR of 41 (30–57) mL/min/1.73 m^2^ (Table 1). Patients taking at least one OAT1/OAT3 inhibitor were significantly older and had a higher BMI than those not taking an OAT1/OAT3 inhibitor. There were no significant intergroup differences in the CKD stages, the time since transplantation, or the proportions of patients treated with cyclosporine or tacrolimus (Table 1). With regard to laboratory parameters, the group of patients with at least one OAT1/OAT3 inhibitor had a significantly lower albumin level and significantly higher CRP (C-reactive protein), glucose, and UT levels than the group not taking an inhibitor.

### 2.2. Plasma Accumulation of UTs in Patients Taking OAT1/OAT3 Inhibitors

**Figure 1:**
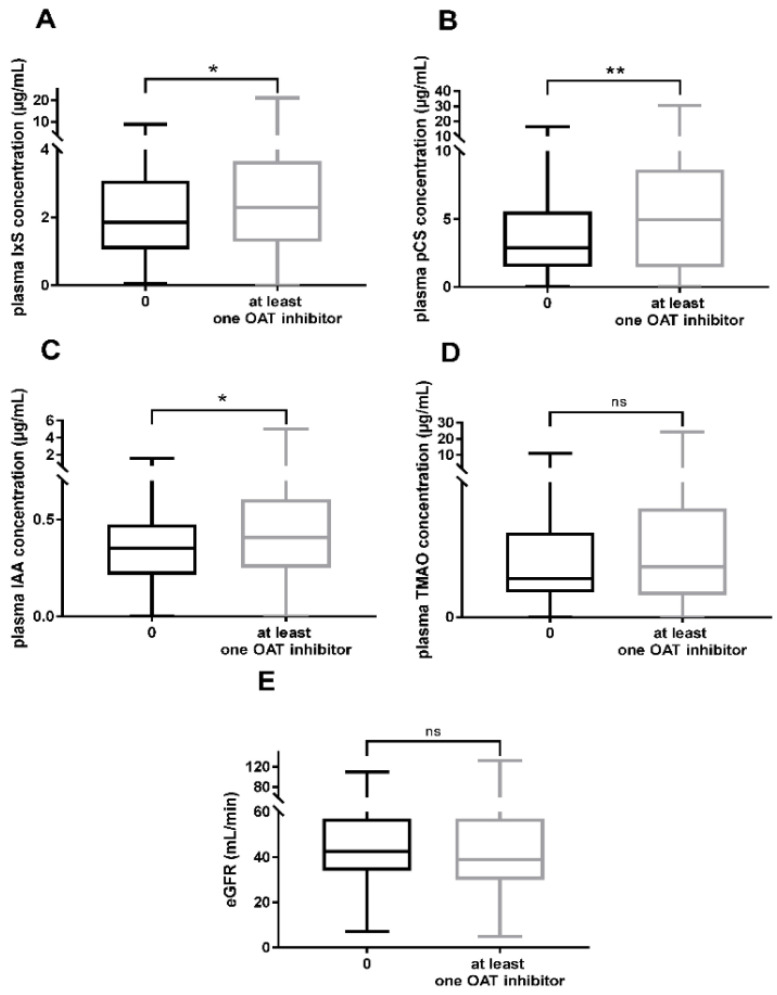
Plasma accumulation of UTs in patients prescribed at least one OAT1/OAT3 inhibitor (*n* = 311) vs. patients not prescribed OAT1/OAT3 inhibitors (*n* = 92) for IxS (**A**), pCS (**B**), IAA (**C**), TMAO (**D**), together with the eGFR (**E**). * *p* < 0.05; ** *p* < 0.01.

We studied the effect of the prescription of at least one OAT1/OAT3 inhibitor on the plasma levels of UTs (IxS, pCS, IAA, and TMAO). The median (IQR) plasma concentrations were significantly higher for patients treated with at least one OAT1/OAT3 inhibitor than for the other patients when considering IxS (2.30 µg/mL (1.29–3.60) vs. 1.86 µg/mL (1.09–3.04), respectively; *p* = 0.030), pCS (4.96 µg/mL (1.57–8.56) vs. 2.90 µg/mL (1.52–5.48), respectively; *p* = 0.008), and IAA (0.41 µg/mL (0.26–0.60) vs. 0.36 µg/mL (0.23–0.47), respectively; *p* = 0.015) (Figure 1A–C). The intergroup difference was not significant for TMAO (Figure 1D). To rule out an effect of renal function on UT accumulation, we compared the two groups of patients with regard to the eGFR; the difference was not statistically significant (Figure 1E).

Next, we studied the effect of the three most frequently prescribed OAT1/OAT3 inhibitors (furosemide, acetylsalicylate, and pantoprazole) on the UT concentrations. Patients treated with furosemide (*n* = 90) had significantly higher levels of TMAO, IAA, pCS, and IxS than patients not treated with furosemide (data not shown). However, furosemide-treated patients also had a significantly lower eGFR than nontreated patients. We, therefore, focused on furosemide-treated and nontreated patients at CKD stages 4 and 5 (*n* = 94), who did not differ with regard to the eGFR (Supplemental Figure S1). The furosemide-treated subgroup had a significantly higher median plasma TMAO level (2.18 µg/mL (1.29–5.14)) than the nontreated subgroup (1.26 µg/mL (0.80–2.33); *p* = 0.012). Lastly, the plasma UT concentrations did not differ in patients treated with pantoprazole (*n* = 159) or those with acetylsalicylate (*n* = 153) vs. patients not taking the corresponding drug (data not shown).

### 2.3. Factors Associated with the Plasma Accumulation of UTs

We first performed a univariate logistic regression, in order to search for factors (including the prescription of an OAT1/OAT3 inhibitor) associated with the plasma concentrations of the UTs (pCS, IxS, and IAA). Patients with an above-median plasma pCS concentration were significantly older and had a higher creatinine level, a lower eGFR, and a higher frequency of at least one prescribed OAT1/OAT3 inhibitor, relative to patients with a below-median plasma pCS concentration (Supplemental Table S1). On the same lines, the univariate regression analysis highlighted an association between the plasma pCS concentration on one hand and age (OR (95%CI) = 1.03 (1.02; 1.05), *p* < 0.0001), eGFR (OR = 0.96 (0.95; 0.97), *p* < 0.0001), time since transplantation (OR = 1.002 (1.000; 1.004), *p* = 0.088), plasma level of albumin (OR = 0.95 (0.91; 1.00), *p* = 0.065) and the intake of at least one OAT1/OAT3 inhibitor (OR = 2.23 (1.38; 3.65), *p* = 0.001) on the other hand (Table 2A). The final multivariate logistic regression analysis showed that after adjustment for eGFR, age, plasma albumin level and time since transplantation, the intake of at least one OAT1/OAT3 inhibitor was still significantly associated with a greater pCS concentration (adjusted OR = 2.11 (1.26; 3.61); *p* = 0.005) (Table 2B).

Patients with an above-median plasma IxS concentration were significantly older and had a higher BMI, a lower eGFR, and albumin level (Supplemental Table S2). Patients with an above-median plasma IAA concentration were significantly older and more likely to be male, with a lower eGFR and a non-significant trend towards taking at least one OAT1/OAT3 inhibitor (Supplemental Table S3). Univariate analyses highlighted associations between plasma IxS or plasma IAA concentrations and the presence of at least one OAT1/OAT3 inhibitor (Table 3A,B). However, associations were not significant after adjustment for age, BMI, time since transplantation, plasma albumin level and eGFR for IxS and adjustment for age, sex, and eGFR for IAA in the final multivariate models (data not shown).

## 3. Discussion

With a view to limit plasma UT accumulation in CKD patients, the potential impact of OAT1/OAT3 inhibitors on the renal elimination of UTs needs to be better understood. Our analysis was focused on kidney transplant recipients whose renal function had deteriorated since transplantation and who were taking an average of 11 drugs, i.e., with a high risk of adverse drug reactions. The objective of the present clinical study was to determine whether OAT1/OAT3 inhibitors commonly prescribed to kidney transplant recipients are associated with differences in plasma UT blood levels.

Our results showed for the first time that after adjustment for eGFR, age, plasma albumin level, and time since transplantation, the prescription of at least one OAT1/OAT3 inhibitor was independently associated with a two-fold elevation of plasma pCS levels. Para-cresyl sulfate is known to be associated with a higher risk of cardiovascular complications and mortality in CKD patients [15,16]. In vitro and in vivo studies have identified a number of pathophysiological factors—notably the production of reactive oxygen species [17] and the induction of inflammation [18]—involved in these effects. In our study, patients taking at least one OAT1/OAT3 inhibitor did not have significantly different systolic blood pressure, diastolic blood pressure, or pulse pressure values than patients not taking these drugs. However, our cross-sectional study was not designed to investigate a putative association between OAT1/OAT3 inhibition and cardiovascular adverse events. Nevertheless, given the apparent link between the prescription of OAT1/OAT3 inhibitors and plasma pCS accumulation, longitudinal studies of the putative association between these drugs and an increase in cardiovascular adverse events are now warranted.

We also assessed UT levels in patients treated (or not) with furosemide, acetylsalicylate, and pantoprazole—the three most frequently prescribed OAT1/OAT3 inhibitors in our cohort. Levels of TMAO, IAA, pCS and IxS were higher in furosemide-treated patients than in patients not treated with this drug. However, we cannot rule out an effect of renal function because the furosemide-treated group had a lower eGFR. The difference in blood TMAO levels between the untreated and treated furosemide groups was still significant when we focused on CKD stage 4–5 patients, who had similar eGFRs. These results are in line with Li et al.’s recent report of an interaction between TMAO and loop diuretics in both clinical and in vivo animal studies. In the clinical study, TMAO concentrations were higher in patients treated with loop diuretics than in nontreated patients. Likewise, furosemide injection in mice abolished tubular TMAO secretion, and the UT accumulated in the plasma and kidney [14]. As the accumulation of TMAO was recently linked to a greater risk of cardiovascular adverse events [19], it would be useful to study the effect of furosemide prescription on TMAO levels in a larger cohort of CKD patients. For acetylsalicylate and pantoprazole, the treated and non-treated patients did not differ significantly in terms of UT levels or the eGFR. Overall, the lack of significant results for the single OAT1/OAT3 inhibitors might be due to a lack of statistical power, since only 90, 153, and 159 of the patients in our study were treated with furosemide, acetylsalicylate, and pantoprazole, respectively. Likewise, an analysis based on the daily drug dose (DDD) is a more refined and more accurate than a qualitative (presence or absence) analysis. However, a statistical analysis of the DDD would require a drug-by-drug approach, and so statistical power would be lost.

Little is known about potential drug–UT interactions that might lead to UT accumulation within the blood and then to systemic adverse events. However, interactions between two drugs (one being a transporter substrate and the other being an inhibitor) are well known and are taken into account in prescribing rules. For example, proton pump inhibitors (PPIs) are well-known OAT inhibitors. In methotrexate-treated patients, the administration of PPIs led to a significant accumulation of methotrexate in the plasma, relative to patients not taking PPIs [20]. Likewise, the administration of the PPI lansoprazole has been linked to a greater incidence of hematologic adverse events in patients treated with pemetrexed—an anticancer drug eliminated by OAT3 [21]. Another in vitro study showed that OAT3’s uptake of enalaprilat is inhibited by several inhibitors, including diclofenac, valsartan, and telmisartan [22]. Hence, one can reasonably hypothesize that OAT1/OAT3 transporters are important targets for drug–UT interactions. However, only a few animal studies of drug—UT interactions for OATs have been described in the literature. Luo et al.’s recent study of nephrectomized rats showed that intravenous injection of the OAT3 inhibitor ciprofloxacin led to a significant (491%) increase in the elimination half-life for IxS and a 71% decrease in IxS clearance [13]. The researchers also observed a 272% increase in the AUC, reflecting increased exposure. Similar results were found by Yu et al. for the intravenous administration of the nonsteroidal anti-inflammatory drugs diclofenac and ketoprofen in rats, with increases of 206% and 278% in the AUC for IxS, respectively, and decreases of 71% and 82% in IxS clearance, respectively [23]. Interestingly, the elimination of UTs from the brain might also be impacted by drug–UT interactions related to OAT1/OAT3, as reported by Othsuki et al.; the researchers showed that brain-to-blood transport of IxS was inhibited by OAT3 inhibitors (cimetidine, acyclovir, and benzylpenicillin) in rats [24]. It is clear that most of the published data on this topic were obtained in preclinical studies; our clinical study is the first to have shown a link between commonly prescribed OAT1/OAT3 inhibitors and the plasma pCS concentration in CKD patients.

Our study also had some limitations. Firstly, our results were obtained in patients who developed CKD after kidney transplantation. CKD patients at stage 4 or 5 before kidney transplantation exhibit much higher plasma UT concentrations; this difference reflects a recovery of renal function after transplantation. Therefore, it would be interesting to repeat our study in a cohort of CKD patients before transplantation. In kidney transplant recipients, it remains to be seen whether the observed pCS accumulation has an impact on the occurrence of adverse events. In any case, longitudinal studies are needed to confirm these results. In fact, our present results do not show that prescription of drugs that inhibit OAT1/OAT3 leads to an increase in UTs but rather indicate that they are associated (at a given point in time) with a significantly higher pCS level after adjustment for confounding factors. Our study’s cross-sectional design prevented us from comparing uremic toxin levels before and after the initiation of OAT1/OAT3 inhibitors. This will be an important aspect to be monitored in future research, to ensure that any significant intergroup differences can be attributed to the drugs used rather than to pre-existing differences. The cross-sectional approach has other limitations. Even though all the drug treatments studied here concerned stable regimens initiated more than five half-lives before inclusion in the study, poor treatment compliance cannot be fully ruled out. However, this point is difficult to control for in a cross-sectional study of many OAT1/OAT3 inhibitors.

Secondly, the study focused on potential drug–UT interactions that might impact UT elimination and not interactions that might impact the other phases of UT toxicokinetics. Hence, we cannot rule out the possible presence of confounding factors. The UTs studied here are metabolites produced from amino acids (tryptophan for IxS and IAA, tyrosine for pCS, and choline for TMAO) during gut microbiota metabolism, prior to absorption across the intestinal barrier and into the portal circulation [25]. Changes in the dietary protein/fiber index or other modifications in the gut microbiota can thus modulate blood UT concentrations in CKD patients [26,27,28]. The non-interventional nature of the present study prevented us from collecting additional information from the patients. The lack of a dietary questionnaire and thus quantification of the dietary protein intake constitute a source of bias, since UT levels are influenced by dietary content. However, in CKD patients, it appears that the difference in plasma UT concentration is not associated with gut-microbiota perturbations but is strongly associated with impaired renal elimination of UTs [29].

PBUTs are also characterized by their strong binding to plasma albumin. Hence, drugs that also bind to albumin with high affinity can compete with PBUTs [30,31]. This drug–UT interaction can be used to improve UT dialysis by increasing the free fractions [32,33]. Some of the drugs assessed in our study bind strongly to albumin but none were associated with a greater plasma pCS level. Liver enzymes (cytochrome 2E1 (CYP2E1), sulfotransferase family 1A member 1 (SULT1A1) and flavin-containing mono-oxygenase 3 (FMO3) can convert UT precursors (indole, p-cresol, and trimethylamine) into the respective UTs (IxS, pCS, and TMAO) [25]; in principle, drugs that induce these enzymes could increase blood UT concentrations. To the best of our knowledge, no prescription drugs induce SULT1A1 or FMO, whereas isoniazid, nicotine, and alcohol are examples of CYP2E1 inducers [34]. In the present study, none of the patients had a prescription for isoniazid. Since the study’s objective was to evaluate the effects on UT concentrations of drugs commonly prescribed in transplant recipients, we did not record the patients’ consumption of alcohol and tobacco. Renal proximal tubular cells express other transporters on their apical membrane, some of which (breast cancer resistance protein (BCRP, also known as ABCG2), and multidrug resistance protein 4 (MRP4, also known as ABCC4) are also involved in the secretion of UTs into the urine [35]. Only a few of the patients in our study were taking MRP4 inhibitors (*n* = 5), and none were taking BCRP inhibitors at therapeutic concentrations. Given that a recent in vivo study observed UT accumulation and lower survival in *Abcg2*-knockout CKD mice (compared with control CKD mice), the same type of study could be considered with drugs that inhibit BCRP and MRP4 [36].

Our study had a number of strengths. Firstly, we used validated, sensitive assays of four UTs known to have harmful effects in kidney transplant recipients. Secondly, our clinical study is the first to have looked at the effect of overall OAT1/OAT3 inhibition on plasma UT concentrations. Our results confirm the importance of better understanding the effects of drugs on UT accumulation, in order to limit the occurrence of adverse events in general and cardiovascular adverse events in particular.

## 4. Conclusions

Prescription drugs known to inhibit OAT1/OAT3 were associated with higher plasma levels of pCS, IxS, and IAA in kidney transplant recipients. Only the higher plasma pCS concentration was still significantly associated with the prescription of at least one OAT1/OAT3 inhibitor after adjustment for age, renal function, plasma albumin level, and time since transplantation. Our study is the first to show that OAT1/OAT3-inhibiting drugs commonly prescribed to kidney transplant recipients can be related to circulating pCS concentrations. Even though these results need to be confirmed in longitudinal studies, our initial results emphasize the importance of understanding the interactions between drugs and UTs (particularly those involving OAT1/OAT3) and thus limiting the plasma accumulation of UTs.

## 5. Materials and Methods

The present results are reported in accordance with the Strengthening the Reporting of Observational Studies in Epidemiology (STROBE) guidelines [37].

### 5.1. Study Design and Participants

The present study was an ancillary analysis of the DRUGTOX study [31]. Briefly, the DRUGTOX study followed up 403 adult patients being monitored after kidney transplantation at Amiens University Medical Center (Amiens, France) and who underwent calcineurin inhibitor therapeutic drug monitoring between 4 August 2019, and 11 March 2020. The main inclusion criteria were age 18 or over, kidney transplantation, and available data on the patient’s prescription medications. Patients with acute graft rejection and/or who refused to participate were not included in the study.

For each patient, we recorded clinical data, laboratory data, the plasma concentrations of UTs (IxS, pCS, IAA, and TMAO), and prescriptions of drugs with OAT1/OAT3 inhibition activity at the time when the UT assay was performed.

The study was registered with the French National Data Commission (Commission Nationale de l’Informatique et des Libertés, Paris, France; registration number: PI2019_843_0060). In line with the French legislation on retrospective analyses of clinical practice, approval by an institutional review board was not required. However, patients were free to refuse to participate. The study was registered at ClinicalTrials.gov (15 July 2021) (NCT04963673).

### 5.2. Study Endpoints

The study’s objective was to determine whether the prescription of OAT1/OAT3 inhibitors was associated with an increase in levels of UTs.

### 5.3. Collected Data

The patients’ demographic characteristics (age and sex) and anthropometric characteristics (body mass index (BMI)) were recorded. The clinical characteristics (time since transplantation, CKD stage, etiology of CKD, blood pressure, and any history of hypertension or liver disease), and laboratory data (the eGFR, and plasma levels of creatinine, calcium, phosphate, uric acid, C-reactive protein (CRP), total protein, albumin, and glucose) were collected from the patients’ medical records. The Modification of Diet in Renal Disease (MDRD) equation was used to calculate the eGFR, and the CKD stages were evaluated according to the Kidney Disease Improving Global Outcomes (KDIGO) classification [38].

### 5.4. Identification of Drugs That Inhibit OAT1/OAT3

We first used the TransPortal database (https://transportal.compbio.ucsf.edu/ accessed on 22 June 2021) to screen the patients’ prescriptions for drugs known to inhibit OAT1/OAT3 [39]. This database lists potential interactions between drugs and cellular transporters such as OATs (i.e., effects on transporter expression or localization, substrates, inhibitors, and drug–drug interactions). For drugs prescribed at the time of the UT assay but which were not listed in the TransPortal database, we searched the PubMed database for potential drug–OAT interactions. If a drug was referenced in PubMed as an OAT1/OAT3 inhibitor, it was added to the list of OAT1/OAT3 inhibitors. Next, for each selected drug, the OAT1 and OAT3 half-maximal inhibitory concentrations (IC_50_s) were compared with the corresponding plasma therapeutic concentration ranges, in order to exclude drugs that were not expected to inhibit OAT1/OAT3 at the usual dose. Drugs with an IC_50_ below or within the therapeutic range were selected for further analysis (Table 4), and drugs with an IC_50_ above the therapeutic concentration range were excluded (Supplemental Table S4A,B) [40,41,42,43,44].

After screening against the TransPortal and PubMed databases, 18 OAT1 inhibitors were found in the patients’ prescriptions. Seven of the 18 drugs were found to be OAT1 inhibitors at the corresponding therapeutic concentration: acetylsalicylate, diclofenac, furosemide, lansoprazole, omeprazole, telmisartan, and valsartan (Table 4). We also identified 20 potential OAT3 inhibitors, 13 of which were likely to inhibit the transporter at therapeutic concentrations: bumetanide, diclofenac, esomeprazole, fenofibrate, fluvastatin, furosemide, gemfibrozil, lansoprazole, losartan, omeprazole, pantoprazole, telmisartan, and valsartan (Table 4).

Regarding the duration of treatment, we checked that the OAT1/OAT3 inhibitor drugs had been prescribed for more than five half-lives at the time of the patient’s inclusion in the study so that the steady state had been reached. Moreover, the dose levels of each OAT1/OAT3 inhibitor were recorded for all the cohort patients, to check that standard doses were being administered. The mean dose levels for each studied OAT1/OAT3 inhibitor are reported in Table 4.

### 5.5. Uremic Toxin Assays

Plasma levels of IxS, pCS, IAA and TMAO were assayed using a liquid chromatography (Shimadzu, Marne-la-Vallée, France)—tandem mass spectrometry (3200 QTRAP™, Sciex, Les Ulis, France) technique [58]. Briefly, UTs were extracted from 50 µL of plasma by adding 200 µL of an ice-cold acetonitrile solution containing a mix of deuterated internal standards. The compounds were separated on a pentafluorophenyl propyl column (5 µm, 50 × 2.1 mm, Restek™, Lisses, France), using a gradient of acetonitrile with 0.1% formic acid and ultrapure water with 0.1% formic acid (flow rate: 0.8 mL/min). The compounds were identified and quantified by multiple reaction monitoring after negative-mode (for IxS and pCS) or positive-mode (for IAA and TMAO) electrospray ionization.

### 5.6. Statistical Analyses

Baseline characteristics were described for all participants. The results were expressed as the median (interquartile range, IQR) or the frequency (percentage). The Mann–Whitney test or a chi-squared test was used for intergroup comparisons. Plasma concentrations of UTs (TMAO, IAA, pCS, and IxS) and the eGFR were compared for patients with at least one OAT1/OAT3 inhibitor vs. no OAT1/OAT3 inhibitors, and for patients with vs. without furosemide, acetylsalicylate, and pantoprazole (the three most frequently prescribed OAT1/OAT3 inhibitors in our cohort).

We used logistic regression models to estimate the odds ratio (OR) (95% confidence interval (CI)) for each factor associated with plasma UT concentrations below or above the median values. Firstly, univariate logistic analyses were performed for variables thought to influence plasma UT concentrations: age, sex, BMI, time since transplantation, albumin level, eGFR, and the prescription of at least one OAT1/OAT3 inhibitor at the time of the UT assay. Variables with *p* > 0.10 in the crude model were removed from the multivariate analysis. Next, a multivariate logistic regression analysis of factors associated with plasma UT concentrations was used to assess whether the prescription of at least one OAT1/OAT3 inhibitor was still associated with the plasma UT concentrations (*p* < 0.05). Due to the collinearity of the UTs tested (data not shown), separate multivariate logistic regression models were built for each UT. Statistical analyses were performed with R software (version 3.5.0, Foundation for Statistical Computing, Vienna, Austria).

## Figures and Tables

**Table 1:** Baseline characteristics of the study population.

	All Patients (*n* = 403)	Patients Not Prescribed OAT1/OAT3 Inhibitors (*n* = 92)	Patients Prescribed at Least One OAT1/OAT3 Inhibitor (*n* = 311)	*p*-Value
Demographic characteristics				
Age (years)	56 (48–66)	52 (43–61)	58 (49–670)	0.002
Males	250 (62.0)	59 (64.1)	191 (61.4)	0.727
BMI (kg/m^2^)	26.5 (23.4–29.7)	24.6 (22.5–28.4)	26.9 (23.9–30.4)	<0.001
Clinical characteristics				
Time since transplantation (months)	78.0 (40.0–158.5)	76.0 (39.8–218.3)	78.0 (40.0–146.5)	0.281
CKD stage				
1	13 (3.2)	1 (1.1)	12 (3.9)	
2	68 (16.9)	17 (18.5)	51 (16.4)	
3A + 3B	228 (56.5)	57 (62.0)	171 (55.0)	0.393
4	84 (20.8)	16 (17.4)	68 (21.9)	
5	10 (2.5)	1 (1.1)	9 (2.9)	
Cause of CKD, n (%)				
Diabetes	20 (4.9)	0 (0)	20 (6.4)	
Vascular disease	26 (6.5)	1 (1.1)	25 (8.0)	
Chronic glomerulonephritis	38 (9.4)	12 (13.0)	26 (8.4)	
Polycystic kidney disease	62 (15.4)	15 (16.3)	47 (15.1)	0.043
Interstitial nephritis	5 (1.2)	1 (1.1)	4 (1.3)	
Autoimmune disorder	72 (17.9)	17 (18.5)	55 (17.7)	
Genetic disorder	45 (11.2)	15 (16.3)	30 (9.6)	
Other causes	135 (33.5)	31 (33.7)	104 (33.4)	
SBP (mmHg)	142 (132–158)	142 (130–156)	142 (132–158)	0.700
DBP (mmHg)	80 (72–87)	81 (74–90)	80 (72–86)	0.111
PP (mmHg)	64 (54–75)	62 (50–71)	64 (54–75)	0.156
Hypertension	392 (97.3)	90 (97.8)	301 (96.8)	1.000
Liver disease	1 (0.3)	0 (0)	1 (0.3)	1.000
Characteristics of calcineurin inhibitors				
Cyclosporine	155 (38.46)	38 (41.30)	117 (37.62)	0.524
Tacrolimus	248 (61.54)	54 (58.70)	194 (62.38)	
Laboratory data				
eGFR (MDRD) (mL/min/1.73 m^2^)	41 (30–57)	42.5 (34–57)	39 (30–57)	0.128
Creatinine (µmol/L)	146 (116–187)	142 (116–177)	148 (116–192)	0.387
Calcium (mmol/L)	2.41 (2.32–2.49)	2.41 (2.33–2.50)	2.41 (2.32–2.49)	0.769
Phosphate (mmol/L)	1.05 (0.91–1.20)	1.03 (0.89–1.13)	1.05 (0.91–1.23)	0.084
Uric acid (µmol/L)	471 (390–565)	483 (399–540)	458 (388–573)	0.906
CRP (mg/L)	3.70 (0.60–9.00)	1.80 (0.30–4.20)	4.50 (0.90–11.20)	0.007
Protein (g/L)	67 (64–70)	67 (63–69)	67 (64–70)	0.984
Albumin (g/L)	38.9 (36.7–41.1)	39.8 (38.0–41.7)	38.8 (36.5–40.9)	0.010
Glucose (mmol/L)	5.4 (4.8–6.3)	5.1 (4.6–5.8)	5.5 (4.9–6.5)	<0.001
Uremic toxins				
IxS (µg/mL)	2.22 (1.22–3.43)	1.86 (1.09–3.04)	2.30 (1.29–3.60)	0.030
PCS (µg/mL)	4.32 (1.52–7.76)	2.90 (1.52–5.48)	4.96 (1.57–8.56)	0.008
TMAO (µg/mL)	0.71 (0.35–1.54)	0.58 (0.38–1.25)	0.75 (0.33–1.61)	0.347
IAA (µg/mL)	0.39 (0.25–0.57)	0.36 (0.23–0.47)	0.41 (0.26–0.60)	0.015

The data are quoted as the median (interquartile range) or the frequency (percentage). BMI, body mass index; CKD, chronic kidney disease; CRP, C-reactive protein; DBP, diastolic blood pressure; eGFR, estimated glomerular filtration rate; IAA, indole-acetic acid; IxS, indoxyl sulfate; pCS, para-cresyl sulfate; PP, pulse pressure; SBP, systolic blood pressure; TMAO, trimethylamine-N-oxide.

**Table 2:** Univariate (**A**) and multivariate (**B**) logistic regression of cofactors associated with the plasma pCS concentration.

**A**		
**Crude Model**		
**Plasma pCS Concentration (µg/mL)**		
	**OR (95%CI)**	***p-*** **Value**
Age (years)	1.03 (1.02; 1.05)	<0.0001
BMI (kg/m^2^)	1.02 (0.98; 1.06)	0.245
Sex (male)	1.25 (0.84; 1.87)	0.276
Time since transplantation (months)	1.002 (1.000; 1.004)	0.088
Albumin (g/L)	0.95 (0.91; 1.00)	0.065
At least one OAT1/OAT3 inhibitor	2.23 (1.38; 3.65)	0.001
eGFR (mL/min)	0.96 (0.95; 0.97)	<0.0001
**B**		
**Plasma pCS Concentration (µg/mL)**		
	**OR (95%CI)**	***p*** **-Value**
Unadjusted		
At least one OAT1/OAT3 inhibitor	2.23 (1.38; 3.65)	0.001
Model 1		
At least one OAT1/OAT3 inhibitor	1.98 (1.21; 3.27)	0.007
Age (years)	1.03 (1.02; 1.05)	0.0002
Model 2		
At least one OAT1/OAT3 inhibitor	1.99 (1.19; 3.35)	0.009
Age (years)	1.02 (1.01; 1.04)	0.005
eGFR (mL/min)	0.96 (0.95; 0.98)	<0.0001
Model 3		
At least one OAT1/OAT3 inhibitor	2.04 (1.22; 3.47)	0.007
Age (years)	1.03 (1.01; 1.05)	0.002
eGFR (mL/min)	0.96 (0.95; 0.97)	<0.0001
Albumin (g/L)	1.04 (0.98; 1.11)	0.151
Model 4		
At least one OAT1/OAT3 inhibitor	2.11 (1.26; 3.61)	0.005
Age (years)	1.03 (1.01; 1.05)	0.003
eGFR (mL/min)	0.96 (0.95; 0.97)	<0.0001
Albumin (g/L)	1.05 (0.99; 1.11)	0.123
Time since transplantation (months)	1.00 (1.00; 1.00)	0.401

BMI, body mass index; eGFR, estimated glomerular filtration rate; OR, odds ratio; pCS, para-cresyl sulfate.

**Table 3:** Univariate logistic regression of cofactors associated with the plasma IxS concentration (**A**) and the plasma IAA concentration (**B**).

**A**		
**Crude Model**		
**Plasma IxS Concentration (µg/mL)**		
	**OR (95%CI)**	***p*-Value**
Age (years)	1.02 (1.01; 1.04)	0.002
BMI (kg/m2)	1.04 (0.09; 0.79)	0.016
Sex (male)	0.82 (0.55; 1.23)	0.336
Time since transplantation (months)	1.00 (1.00; 1.01)	0.011
Albumin (g/L)	0.93 (0.88; 0.98)	0.004
At least one OAT1/OAT3 inhibitor	1.57 (0.98; 2.52)	0.062
eGFR (mL/min)	0.92 (0.90; 0.93)	<0.0001
**B**		
**Crude Model**		
**Plasma IAA Concentration (µg/mL)**		
	**OR (95%CI)**	***p*-Value**
Age (years)	1.01 (1.00; 1.03)	0.015
BMI (kg/m2)	1.02 (0.98; 1.06)	0.330
Sex (men)	1.62 (1.08; 2.43)	0.021
Time since transplantation (months)	1.00 (0.69; 1.29)	0.653
Albumin (g/L)	1.02 (0.98; 1.08)	0.304
At least one OAT1/OAT3 inhibitor	1.57 (0.98; 2.52)	0.062
eGFR (mL/min)	0.98 (0.97; 0.99)	<0.0001

BMI, body mass index; eGFR, estimated glomerular filtration rate; IAA, indole acetic acid; IxS, indoxyl sulfate; OR, odds ratio.

**Table 4:** Drugs with an IC_50_s for OAT1/OAT3 below or within therapeutic range, as prescribed to patients in the present study.

Drug	Number of Prescriptions	Mean Dose Level (mg per Day) (Min–Max)	OAT Concerned by the Inhibition	In Vitro Data on OAT1/OAT3 Inhibition			Plasma Therapeutic Concentration Range (µg/mL)	References
				Cell System	Substrate Used	IC_50_ (µg/mL)		
Acetyl salicylate	153	91 (37.5–300)	OAT3	OAT3-HEK293	Cilostazol	2.3	3.2–5.1	[45,46]
Bumetanide	2	6.5 (3–10)	OAT3	S2-hOAT3	Estrone 3-sulfate	0.27–2.80	0.03–0.40	[40,41,47]
Diclofenac	1	NA	OAT1	S2-hOAT1	Para-aminohippurate	1.3	0.5–3.0	[42,48]
			OAT3	S2-hOAT3	Estrone 3-sulfate	2.3		
Esomeprazole	45	23 (20–80)	OAT3	OAT3-HEK293	Methotrexate	0.41	0.78–1.07	[20,49]
Fenofibrate	3	111 (67–200)	OAT3	CHO-OAT3	Sitagliptin	0.8	5–30	[42,50]
Fluvastatin	6	53 (20–80)	OAT3	S2-hOAT3	Estrone 3-sulfate	0.24	0.05–0.40	[42,51]
Furosemide	90	68.5 (20–750)	OAT1	S2-hOAT1	Para-aminohippurate	5.9	2–10	[42,47]
			OAT3	S2-hOAT3	Estrone 3-sulfate	0.56		[42,50]
				CHO-OAT3	Sitagliptine	2.4		[42,47]
Gemfibrozil	1	450	OAT3	S2-hOAT3	Pravastatin	1.7	25	[42,52]
Lansoprazole	7	22.5 (15–30)	OAT1	OAT1-HEK293	Para-aminohippurate	2.8	1.1–3.6	[20,53,54]
			OAT3	OAT3-HEK293	Methotrexate	0.15–0.42		
Losartan	2	50	OAT3	OAT3-HEK293	Uric acid	0.68	0.20–0.65	[42,55]
Omeprazole	32	18.5 (10–20)	OAT1	OAT1-HEK293	Para-aminohippurate	1.7	0.05–4.00	[20,42,53]
			OAT3	OAT3-HEK293	Methotrexate	2.1–2.6		
Pantoprazole	159	25 (10–80)	OAT3	OAT3-HEK293	Methotrexate	1.7	2.0–4.6	[42,53]
Telmisartan	2	40	OAT1	OAT1-HEK293	Uric acid	0.24	0.03–0.37	[56,57]
			OAT3	OAT3-HEK293		0.82		
Valsartan	9	124.5 (40–160)	OAT1	OAT1-HEK293	Uric acid	7	0.8–6.0	[42,56]
			OAT3	OAT3-HEK293		0.87		

## Data Availability

Data sharing is not applicable to this article.

## Supplementary Materials

The following are available online at https://www.mdpi.com/article/10.3390/toxins14010015/s1, Figure S1: Plasma accumulation of IxS (A), pCS (B), IAA (C) and TMAO (D), and the eGFR (E) in CKD stage 4-5 patients treated (*n* = 37) or not (*n* = 57) with furosemide, Table S1: Characteristics of the study population, as a function of the median plasma pCS concentration, Table S2: Characteristics of the study population, as a function of the median plasma IxS concentration, Table S3: Characteristics of the study population, as a function of the median plasma IAA concentration. Table S4: Drugs with an IC50s for OAT1 (A) or OAT3 (B) above the therapeutic range, as prescribed to patients in the present study.
