# Supplementary material for: The Prescription of Drugs That Inhibit Organic Anion Transporters 1 or 3 Is Associated with the Plasma Accumulation of Uremic Toxins in Kidney Transplant Recipients
Source: Toxins (Basel). 2021 Dec 25;14(1):15. doi: 10.3390/toxins14010015 (PMC8780284; doi:10.3390/toxins14010015)
Supplement: Supplementary file 1 [file toxins-14-00015-s001.zip › toxins-1492809-supplementary.pdf]

Supplementary Materials: The Prescription of Drugs that Inhibit Organic Anion Transporters 1 or 3 is Associated with the Plasma Accumulation of Uremic Toxins in Kidney Transplant Recipients

Camille André, Touria Mernissi, Gabriel Choukroun, Youssef Bennis, Saïd Kamel, Sophie Liabeuf† and Sandra Bodeau†*

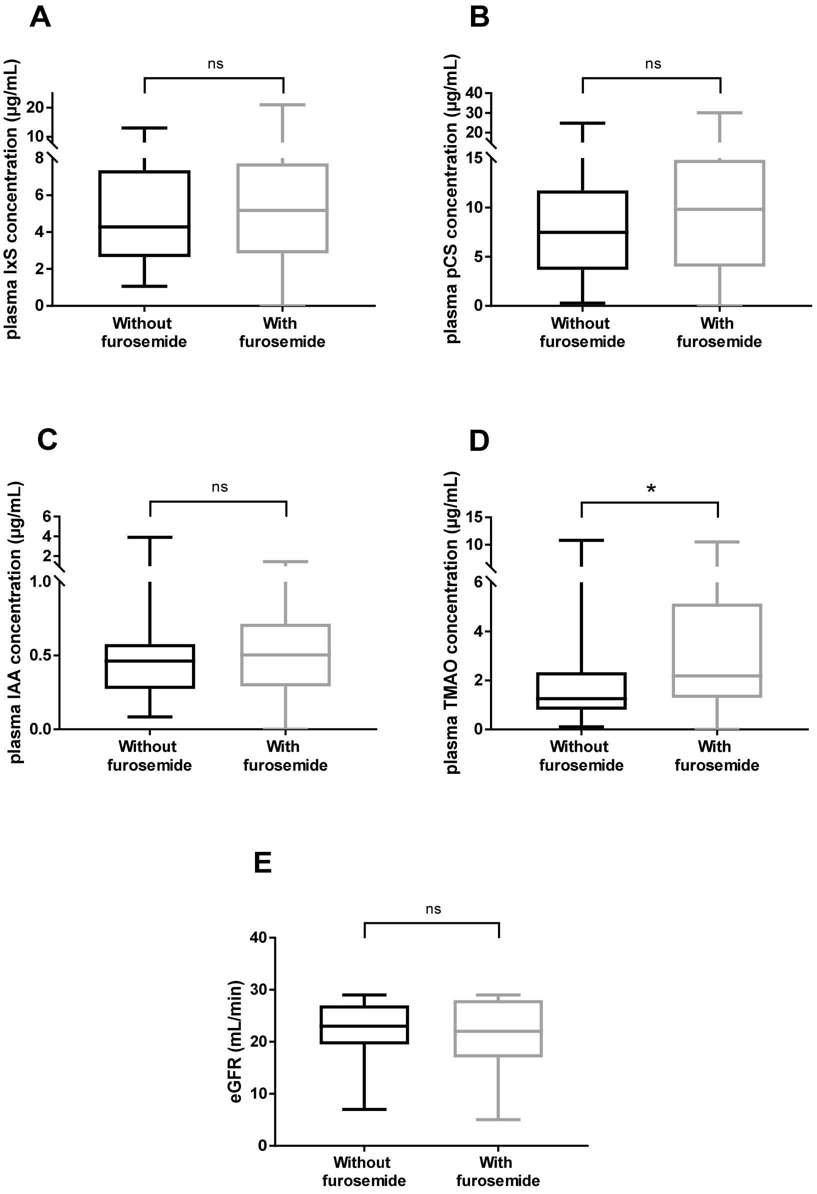

Figure S1. Plasma accumulation of IxS (A), pCS (B), IAA (C) and TMAO (D), and the eGFR (E) in CKD stage 4/5 patients treated (n=37) or not (n=57) with furosemide. *$p<0.05$

Table S1. Characteristics of the study population, as a function of the median plasma pCS concentration.

	**All patients (*n*=403)**	**pCS<4.32 μg/mL (*n*=202)**	**pCS > 4.32 μg/mL (*n*=201)**	***p*-Value**
Demographic characteristics				
Age (years)	56 (48-66)	53 (44-63)	59 (51-69)	< 0.0001
Males	250 (62.03)	120 (59.41)	130 (64.68)	0.3234
BMI (kg/m^2)	26.5 (23.4-29.7)	25.6 (22.9-29.8)	26.8 (24.1-29.5)	0.1834
Clinical characteristics				
Time since transplantation (months)	78.0 (40.0-158.5)	74.0 (36.0-149.8)	82.0 (43.0-167.0)	0.1171
CKD stage				
1	13 (3.23)	12 (5.94)	1 (0.5)	
2	68 (16.87)	46 (22.77)	22 (10.95)	
3A + 3B	228 (56.58)	118 (58.42)	110 (54.73)	< 0.0001
4	84 (20.84)	25 (12.38)	59 (29.35)	
5	10 (2.48)	1 (0.5)	9 (4.48)	
Cause of CKD				
Diabetes	20 (4.96)	7 (3.47)	13 (6.47)	
Vascular disease	26 (6.45)	13 (6.44)	13 (6.47)	
Chronic glomerulonephritis	38 (9.43)	20 (9.90)	18 (8.96)	
Polycystic kidney disease	62 (15.38)	29 (14.36)	33 (16.42)	
Interstitial nephritis	5 (1.24)	2 (0.99)	3 (1.49)	0.9323
Autoimmune disorder	72 (17.87)	36 (17.82)	36 (17.91)	
Genetic disorder	45 (11.17)	23 (11.39)	22 (10.95)	
Other causes	106 (26.30)	56 (27.72)	50 (24.8)	
More than one cause	29 (7.20)	16 (7.92)	13 (6.47)	
SBP (mmHg)	142 (132-158)	141 (131-155)	142.5 (133-160)	0.2681
DBP (mmHg)	80 (72-87)	80 (72-87)	80 (73-87)	0.7741
PP (mmHg)	64 (54-75)	62 (53-75)	66 (54-75)	0.2668
Hypertension	392 (97.30)	194 (96.04)	197 (98.01)	0.3804
Liver disease	1 (0.25)	0 (0)	1 (0.50)	0.4988
Laboratory data				
eGFR (MDRD) (ml/min/1.73m^2)	41 (30-57)	49 (34.3-61)	36 (27-47)	< 0.0001
Creatinine (μmol/L)	146 (116-187)	126 (99-161)	166 (127-214)	< 0.0001
Calcium (mmol/L)	2.41 (2.32-2.49)	2.42 (2.33-2.49)	2.40 (2.30-2.48)	0.1245
Phosphate (mmol/L)	1.05 (0.91-1.20)	1.00 (0.87-1.14)	1.09 (0.97-1.24)	< 0.0001
Uric acid (μmol/L)	471 (390-565)	453 (387-550)	485 (392-577)	0.1305
CRP (mg/L)	3.70 (0.60-9.00)	3.45 (0.96-9.65)	3.77 (0.55-8.90)	0.9376
Protein (g/L)	67 (64-70)	67 (65-70)	66 (63-70)	0.1262
Albumin (g/L)	38.9 (36.7-41.1)	39.2 (37.5-41.4)	38.7 (36.4-40.9)	0.0311
Glucose (mmol/L)	5.4 (4.8-6.3)	5.4 (4.8-6.3)	5.4 (4.9-6.3)	0.9392
OAT1/OAT3 inhibitors				
Number of OAT1/OAT3 inhibitors				
0	92	60	32	
1	145	68	77	
2	129	64	65	0.0011
3	34	9	25	
4	3	1	2	
at least one OAT inhibitor	311	142	169	0.0015

The data are quoted as the median (interquartile range) or the frequency (percentage). BMI, body mass index; CKD, chronic kidney disease; CRP, C-reactive protein; DBP, diastolic blood pressure; eGFR, estimated glomerular filtration rate; MDRD, Modification of Diet in Renal Disease; pCS, para-cresyl sulfate; PP, pulse pressure; SBP, systolic blood pressure.

Table S2. Characteristics of the study population, as a function of the median plasma IxS concentration.

	**All patients (*n*=403)**	**pCS<4.32 µg/mL (*n*=202)**	**pCS > 4.32 µg/mL (*n*=201)**	***p*-Value**
Demographic characteristics				
Age (years)	56 (48-66)	54 (44-65)	58 (50-68)	0.0039
Males	250 (62.03)	130 (64.36)	120 (59.70)	0.3897
BMI (kg/m²)	26.5 (23.4-29.7)	25.6 (22.8-29.0)	27.3 (23.9-30.7)	0.0066
Clinical characteristics				
Time since transplantation (months)	78.0 (40.0-158.5)	69.5 (32.0-144.8)	87.0 (48.0-168.0)	0.0088
CKD stage				
1	13 (3.23)	13 (6.44)	0 (0)	< 0.0001
2	68 (16.87)	60 (29.70)	8 (3.98)	
3A + 3B	228 (56.58)	116 (57.43)	112 (55.72)	
4	84 (20.84)	13 (6.44)	71 (35.32)	
5	10 (2.48)	0 (0)	10 (4.98)	
Cause of CKD				
Diabetes	20 (4.96)	10 (4.95)	10 (4.98)	0.9405
Vascular disease	26 (6.45)	10 (4.95)	16 (7.96)	
Chronic glomerulonephritis	38 (9.43)	17(8.42)	21 (10.45)	
Polycystic kidney disease	62 (15.38)	31 (15.35)	31 (15.42)	
Interstitial nephritis	5 (1.24)	2 (0.99)	3(1.49)	
Autoimmune disorder	72 (17.87)	39(19.31)	33 (16.42)	
Genetic disorder	45 (11.17)	23 (11.39)	22 (10.95)	
Other causes	106 (26.30)	56 (27.72)	50 (24.88)	
More than one cause	29 (7.20)	14 (6.93)	15 (7.46)	
SBP (mmHg)	142 (132-158)	140 (131-154)	144 (133-160)	0.0836
DBP (mmHg)	80 (72-87)	80 (72-87)	80 (72-87)	0.4387
PP (mmHg)	64 (54-75)	62 (52-70)	66 (55-79.8)	0.0169
Hypertension	392 (97.30)	195 (96.53)	196 (97.51)	0.7710
Liver disease	1 (0.25)	0 (0)	1 (0.50)	0.4988
Laboratory data				
eGFR (MDRD) (ml/min/1.73m²)	41 (30-57)	55 (41-64)	33 (24-40)	< 0.0001
Creatinine (µmol/L)	146 (116-187)	121 (98-148)	177 (146-228)	< 0.0001
Calcium (mmol/L)	2.41 (2.32-2.49)	2.43 (2.34-2.49)	2.39 (2.30-2.49)	0.1342
Phosphate (mmol/L)	1.05 (0.91-1.20)	1.00 (0.87-1.12)	1.12 (0.98-1.29)	< 0.0001
Uric acid (µmol/L)	471 (390-565)	450 (370-531)	499 (406-597)	0.0008
CRP (mg/L)	3.70 (0.60-9.00)	2.34 (0.44-10.82)	4.39 (0.68-8.68)	0.2193
Protein (g/L)	67 (64-70)	67 (65-70)	66 (63-70)	0.1958
Albumin (g/L)	38.9 (36.7-41.1)	39.3 (37.5-41.6)	38.6 (36.3-40.8)	0.0010
Glucose (mmol/L)	5.4 (4.8-6.3)	5.4 (4.8-6.1)	5.4 (4.9-6.3)	0.6105
OAT1/OAT3 inhibitors				
Number of OAT1/OAT3 inhibitors				
0	92	54	38	0.0146
1	145	76	69	
2	129	62	67	
3	34	10	24	
4	3	0	3	
at least one OAT inhibitor	311	148	163	0.0796

The data are quoted as the median (interquartile range) or the frequency (percentage). BMI, body mass index; CKD, chronic kidney disease; CRP, C-reactive protein; DBP, diastolic blood pressure; eGFR, estimated glomerular filtration rate; IxS, indoxyl sulfate; MDRD, Modification of Diet in Renal Disease; PP, pulse pressure; SBP, systolic blood pressure.

Table S3. Characteristics of the study population, as a function of the median plasma IAA concentration.

	**All patients (*n*=403)**	**pCS<4.32 μg/mL (*n*=202)**	**pCS > 4.32 μg/mL (*n*=201)**	***p*-Value**
Demographic characteristics				
Age (years)	56 (48-66)	54 (46-64)	58 (49-68)	0.0148
Males	250 (62.03)	114 (56.44)	136 (67.66)	0.0265
BMI (kg/m^2)	26.5 (23.4-29.7)	26.2 (22.8-29.7)	26.5 (23.8-29.7)	0.2869
Clinical characteristics				
Time since transplantation (months)	78.0 (40.0-158.5)	75.0 (34.5-159.8)	79.0 (43.0-157.0)	0.6069
CKD stage				
1	13 (3.23)	10 (4.95)	3 (1.49)	
2	68 (16.87)	40 (19.80)	28 (13.93)	0.0035
3A + 3B	228 (56.58)	119 (58.91)	109 (54.23)	
4	84 (20.84)	31 (15.35)	53 (26.37)	
5	10 (2.48)	2 (0.99)	8 (3.98)	
Cause of CKD				
Diabetes	20 (4.96)	9 (4.46)	11 (5.47)	
Vascular disease	26 (6.45)	11 (5.45)	15 (7.46)	
Chronic glomerulonephritis	38 (9.43)	21 (10.40)	17(8.46)	
Polycystic kidney disease	62 (15.38)	27 (13.37)	35 (17.41)	
Interstitial nephritis	5 (1.24)	3 (1.49)	2 (1.00)	0.9306
Autoimmune disorder	72 (17.87)	38 (18.81)	34 (16.92)	
Genetic disorder	45 (11.17)	23 (11.39)	22 (10.95)	
Other causes	106 (26.30)	54 (26.73)	52 (25.87)	
More than one cause	29 (7.20)	16 (7.92)	13 (6.47)	
SBP (mmHg)	142 (132-158)	143 (134-155)	141 (130-159)	0.7565
DBP (mmHg)	80 (72-87)	80 (74-88)	80.0 (71-87)	0.4087
PP (mmHg)	64 (54-75)	63 (55-74)	64 (52-76)	0.9621
Hypertension	391 (97.30)	198 (98.02)	193 (96.02)	0.2590
Liver disease	1 (0.25)	1 (0.50)	0 (0)	1.0000
Laboratory data				
eGFR (MDRD) (ml/min/1.73m^2)	41 (30-57)	46 (37-59)	37 (28-52)	< 0.0001
Creatinine (μmol/L)	146 (116-187)	134 (108-171)	159 (125-206)	< 0.0001
Calcium (mmol/L)	2.41 (2.32-2.49)	2.42 (2.33-2.49)	2.40 (2.31-2.49)	0.1476
Phosphate (mmol/L)	1.05 (0.91-1.20)	1.03 (0.90-1.17)	1.07 (0.93-1.24)	0.0382
Uric acid (μmol/L)	471 (390-565)	456 (374-547)	485 (397-579)	0.0678
CRP (mg/L)	3.70 (0.60-9.00)	3.38 (0.55-7.90)	3.97 (0.63-10.99)	0.3693
Protein (g/L)	67 (64-70)	67 (64-70)	67 (64-70)	0.7404
Albumin (g/L)	38.9 (36.7-41.1)	38.9 (36.6-41.0)	39.0 (36.8-41.2)	0.6791
Glucose (mmol/L)	5.4 (4.8-6.3)	5.3 (4.8-6.3)	5.5 (4.9-6.3)	0.4898
OAT1/OAT3 inhibitors				
Number of OAT1/OAT3 inhibitors				
0	92	54	38	
1	145	71	74	
2	129	60	69	0.3587
3	34	15	19	
4	3	2	1	
at least one OAT inhibitor	311	148	163	0.0796

The data are quoted as the median (interquartile range) or the frequency (percentage). BMI, body mass index; CKD, chronic kidney disease; CRP, C-reactive protein; DBP, diastolic blood pressure; eGFR, estimated glomerular filtration rate; IxS, indoxyl sulfate; MDRD, Modification of Diet in Renal Disease; PP, pulse pressure; SBP, systolic blood pressure.

Table S4. Drugs with an IC_{50}s for OAT1 (A) or OAT3 (B) above the therapeutic range, as prescribed to patients in the present study.

A

OAT1		
Drug	IC_{50} (µg/mL)	Plasma therapeutic concentration range (µg/mL)
Bumetanide	2.7	0.03-0.40 [40,41]
Candesartan	7.4	0.08-0.18 [42]
Fluvastatin	10.8	0.05-0.40 [42]
Hydrochlorothiazide	20.03	0.04-2.00 [42]
Ketoconazole	169.5	1-6 [42]
Losartan	5.07	0.20-0.65 [42,55]
Pantoprazole	24.2	4.6 [42]
Paracetamol	96.1	5-25 [42]
Pravastatin	173.2	0.05-0.15 [43]
Rifampicin	65.1	0.1-10 [42]
Simvastatin	30.8	0.005-0.015 [42]

B

OAT3		
Drug	IC_{50} (µg/mL)	Plasma therapeutic concentration range (µg/mL)
Candesartan	1.3	0.08-0.18 [42]
Cimetidine	43.6	0.25-3.00 [42]
Hydrochlorothiazide	280.4	0.04-2.00 [42]
Indapamide	4.02	0.17-0.27 [44]
Pravastatin	5.8	0.05-0.15 [43]
Simvastatin	13.5	0.005-0.015 [42]
Sitagliptin	65.1	0.05-038 [42]
